# Determinants of acceptance of Coronavirus disease-2019 (COVID-19) vaccine among Lebanese health care workers using health belief model

**DOI:** 10.1371/journal.pone.0264128

**Published:** 2022-02-22

**Authors:** Dalal Youssef, Linda Abou-Abbas, Atika Berry, Janet Youssef, Hamad Hassan

**Affiliations:** 1 Preventive Medicine Department, Ministry of Public Health, Beirut, Lebanon; 2 Bordeaux Research Center for Population Health, Institut de santé publique, d’épidémiologie et de développement (ISPED), Bordeaux University, Bordeaux, France; 3 Clinical trial Program, Ministry of Public Health, Beirut, Lebanon; 4 Neuroscience Research Center, Faculty of Medical Sciences, Lebanese University, Beirut, Lebanon; 5 Al Zahraa hospital University Medical Center, Beirut, Lebanon; 6 Ministry of Public Health, Beirut, Lebanon; Chinese Academy of Medical Sciences and Peking Union Medical College, CHINA

## Abstract

Since Health care workers (HCWs) are at high occupational risk for COVID-19, they are prioritized for immunization. This study aimed to assess the acceptance rate of the COVID-19 vaccine among HCWs and to identify its determinants. A web-based cross-sectional study was conducted between10 and 31 December 2020 among Lebanese HCWs. The Health Belief Model (HBM) was used as a theoretical framework. Multivariable logistic analyses were carried out to identify the factors associated with the acceptance of the COVID-19 vaccine among HCWs. A total of 1800 HCWs have completed the survey. Around half (58.10%) of them were frontline HCWs and aged between (30–49) years old. Over two-thirds (67.33%) of the participants have received the seasonal influenza vaccine. The acceptance rate of the COVID-19 vaccine among surveyed HCWs was 58%. HCWs who were male (aOR = 1.99, 95% CI (1.41–2.80)), working in the frontlines (aOR = 1.61, 95% CI (1.17–2.21), and those who have received influenza vaccination for the current year (aOR = 1.38, 95% CI(0.99–1.92)) were more willing to get the COVID-19 vaccine. However, factors such as living in rural areas (aOR = 0.61, 95% CI (0.44–0.84)), and being previously diagnosed with COVID-19 (aOR = 0.66, 95%CI (0.45–0.96) were found negatively associated with vaccine acceptance. In terms of health beliefs items, concerns related to the novelty of vaccine (aOR = 0.42, 95% CI (0.25–0.71)), side effects/vaccine safety (aOR = 0.41, 95% CI (0.23–0.73), reliability of manufacturer (aOR = 0.43, 95% CI (0.30–0.63)), and the number of required doses (aOR = 0.58, 95% CI (0.40–0.84)) were also negatively associated with the willingness to get vaccinated against COVID-19. Remarkably, concerns such as the limited accessibility (aOR = 1.68, 95% CI (1.14–2.47)), and availability of vaccines (aOR = 2.16, 95% CI (1.46–3.20)) were associated with an increased likelihood of willingness to receive the COVID-19 vaccine. With regards to cues of action, receiving reliable and adequate information about the vaccine (aOR = 1.98, 95% CI (1.36–2.88)), recommendation by health authorities (aOR = 1.93, 95% CI(1.33–2.81)), and recommendations from health facilities (aOR = 2.68, 95% CI(1.80–3.99)) were also positively associated with vaccine acceptance. Lastly, perception of COVID-19 vaccine benefits by HCWs in terms of protecting them and their close contacts (patients, family members, and friends) from COVID-19 infection (aOR = 4.21, 95% CI (2.78–7.11)) was associated with an increased likelihood of vaccine uptake. The moderate acceptance rate of the COVID-19 vaccine among HCWs found in our study could have broader extents. Understanding and pointing out factors impairing vaccine acceptance such as concerns about the novelty of vaccine and manufacturers’ reliability are required to reach a higher vaccination rate.

## Introduction

Coronavirus disease 2019 (COVID-19) has instigated a public health and economic calamity worldwide [1]. It continues to inflict substantial morbidity and mortality despite intervention efforts. As of 20 January 2021, there have been over 85 million cases and 1.8 million deaths reported [2]. To control the blowout of COVID-19, countries have focused their efforts on slowing the spread of the disease by adopting a variety of non-pharmaceutical interventions, including travel restriction, schools’ closure, remote schooling, forced quarantine, and lockdown [3]. Despite that these measures are considered essential in the short term, a long-lasting solution represented by vaccines is urgently needed to dramatically lessen the mortality burden and conceivably halt local transmission [4].

In response to this need, numerous pharmaceutical companies were proactive since the early phase of the pandemic, as they took the initiative to launch vaccine development as soon as the first Severe Acute Respiratory Syndrome Coronavirus (SARS-CoV-2) genome sequence was published [5]. Consequently, several prophylactic vaccines against COVID-19 are being developed including mRNA-based vaccines, DNA-based vaccines, inactivated, live attenuated, sub-unit, and replicating or non-replicating viral vector-based vaccines were developed across multiple countries [6]. Fortunately, the rapid development of many promising vaccines was crowned in the final weeks of 2020 by the first authorization and shipping of doses [7]. Many countries have authorized respectively the emergency use of COVID-19 vaccines [8]. However, limited COVID-19 vaccine supplies, combined with wide disparities in transmission dynamics and SARS-COV-2 infection severity between groups, will be extremely challenging for the first several months of the vaccination campaign [9]. Accordingly, it urges the need to establish an effective policy for COVID-19 vaccine allocation based on a deep understanding of the current epidemiological characteristics of COVID-19 [10]. This information includes key factors of group heterogeneity such as susceptibility to get COVID-19, the severity of outcomes, and contact rates. The sharp variances in the epidemiology of influenza viruses and SARS-Cov2, hamper the ability to use the existing influenza vaccination policies as a mirror for the vaccination against COVID-19. For example, there is lower susceptibility to infection among children and adolescents, and a substantially higher fatality rate that increases evidently with age is associated with COVID-19 [11].

Given HCW’s high occupational risk of being infected by SARS-Cov2, especially those working in the frontline, hence protecting them against COVID-19 is a top priority [12]. Once infected by SARS-COV-2, HCWs had the potential of becoming “super-spreaders” and they are likely to spread the virus to their patients, residents of health facilities, loved ones, and members of the community. Furthermore, the sickness of HCWs impacted negatively the medical services provided by the healthcare facilities [13].

Therefore, the early access of HCWs to the COVID-19 vaccines is critical to ensuring their health and safety, thus protecting their patients, families, communities, and the broader health of the population. Based on the Values Framework and Population Prioritization Roadmap, issued by a World Health Organization (WHO) advisory group on immunization, countries that plan to roll out COVID-19 should prioritize health care workers (HCWs) and other at-risk populations for vaccination [14]. Of note, several studies have shown that not all HCWs are willing to use COVID-19 vaccines when they become accessible in their country. Concerns about vaccination safety and side effects, as well as the rapid pace of COVID-19 vaccines development and approval, could lead to COVID-19 vaccines hesitancy [7, 14, 15].

In Lebanon, since the early phase of vaccine development, national efforts were engrossed in securing the country’s portion of the COVID-19 vaccine through negotiation conducted with Pfizer/BioNTech Company. The vaccine is intended to reach Lebanon by mid-February 2021 and the expectable supply will cover 15% of the population. The vaccine will be provided for free for the population following a voluntary process. In addition, the COVAX platform will cover 20% of the population. As of March 2, 2021, there were more than 2567 confirmed COVID-19 cases and 45 deaths reported among HCWs in Lebanon [16]. Similarly, to other countries and based on the current epidemiological data, the Ministry of Public Health (MOPH) has prioritized the COVID-19 vaccination for all HCWs. Hence, the government must start to gauge current levels of HCWs’ willingness to receive a potentially safe and effective COVID-19 vaccine and to identify determinants of acceptance and refusal of the vaccine. In the light of the current flood of misinformation regarding the vaccine’s safety, examining HCWs’ attitudes toward COVID-19 vaccination could enable researchers and policymakers in developing effective strategies and planning relevant interventions ahead of time. This could minimize vaccine hesitancy among HCWs.

The objectives of this study were to gauge the acceptance rate of the COVID-19 vaccine among HCWs. It also aimed to assess the health belief model (HBM) in terms of perceived susceptibility, severity, benefits, and barriers and to appraise participants’ cues to action and self-efficacy. In addition, we sought to identify the determinants of HCW’s COVID-19 vaccine acceptance.

## Methods

### Study tool and design

A cross-sectional study, using an online survey, was conducted during the early phase preceding the arrival of the COVID-19 vaccine in Lebanon and the launch of the national vaccination plan between 10th and 31st December 2020. As the Lebanese government recommended the public minimalize face-to-face interaction, potential respondents were electronically invited to participate.

### Questionnaire development

An extensive review of the literature was conducted to list available resources on acceptance of vaccines, as well as to identify relevant items and scales on vaccination. The Health Belief Model (HBM) was used extensively in vaccination research to study vaccination behaviors and to identify participant perceptions towards disease and vaccination. In the present study, it was adopted as a theoretical framework to assess HCWs’ drives for receiving the COVID-19 vaccine [17]. It comprises six key domains which impact willingness to vaccinate: perceived susceptibility to COVID-19, perceived severity of COVID-19 infection, perceived benefits of COVID-19 vaccine, perceived barriers of COVID-19 vaccine, cues to action, and self-efficacy [18].

A 72-item structured questionnaire was initially developed and designed by the authors to cover important aspects of COVID-19 vaccination among HCWs based on the HBM (S1 File). A panel of experts has reviewed the developed questionnaire and assessed the clarity of the questions, interpretability, and accuracy of the domains. Then, the original English draft of the questionnaire was translated and adapted to the Arabic language based on the standard translation guidelines [19]. The questionnaire was pre-tested among 20 HCWs for assessing survey flow, functionality, readability, comprehension of instructions, and clarity. Based upon feedback from the pre-test, minor modifications in terms of readability and clarity were made to the questionnaire. The questionnaire’s reliability was also tested, and the Cronbach Alpha value was 0.82. The average time for completing the survey was 8 minutes. The questionnaire was self-administered and its final version consisted of open-ended questions and was divided into four main sections:

1. **The baseline characteristics of the study participants section** included information about age, gender, marital status, urbanicity, specialty, place of work, clinical experience, health status, underlying health conditions, and health coverage of the participants. Surveyed HCWs were also asked whether they had a previous history of COVID-19 or they had one of their family members or colleagues had previously been infected with COVID-19. Their past behavior toward vaccination was also explored. This included their influenza vaccine intake and their refusal of any kind of vaccine in the past.
2. **Willingness to receive the COVID-19 vaccine section** was assessed using one question: Will you take Covid-19 vaccine once it is available?” with response categories of “Yes” and “No”. HBM constructs section which included six domains:
  - **The perceived susceptibility domain** consisted of four questions addressing HCW’s sights about their possible risk of getting infected by COVID-19 [20].
  - **The perceived severity domain** consisted of five questions that relate to the patient’s concerns about the seriousness of COVID-19.
  - **The perceived benefits domain** comprised seven questions linked to the perceived positive outcomes of getting vaccinated against COVID-19 in terms of reducing their susceptibility to contracting the illness or the severity of symptoms if being infected by COVID-19 [21].
  - The **perceived barriers** domain consisted of 13 questions that pinpointed the patient’s concerns or negative beliefs toward COVID-19 vaccines. Of note, Responses to questions related to perceived susceptibility, severity, benefits, and barriers were graded on a 3-point Likert scale, an agreement scale ranging from ‘1’ for the “disagree” to ‘3’ for the “agree” responses.
  - **The cues to action domain** comprised seven questions addressing different clues or recommendations that promote the willingness of HCW to get vaccinated against COVID-19. Responses to these statements were ranked on a 3-point Likert scale ranging from ‘1’ for the “No” response, ‘2’ for “Not sure” and ‘3’ for the response “Yes”.
  - **The self-motivation domain** comprised 2 statements that addressed HCW’s willingness to improve his health, e.g., adopting a healthy lifestyle. Responses to questions related to self-motivation were graded on a 3-point Likert scale, an agreement scale ranging from ‘1’ for never to ‘3’ all the time [22].
3. **Knowledge about vaccine section** consisted of 8 questions assessing awareness of HCWs about COVID-19 vaccines. All the items were answered on a true/false basis and an additional “do not know” option. A correct response had a value of ‘1′ and a "wrong" or don’t know response had a value of ‘0′. Hence, the aggregate score for all 8 knowledge questions would range from 0 to 8 points. Participants ‘overall knowledge was categorized using modified Bloom’s cut-off point, as good if the score was >60% (5–8 points), and poor if the score was less than 60% (< 5 points).

Participants were also requested to rank the reliability of used information sources about the COVID-19 vaccine as well.

### Sample size calculation

To calculate the sample size of the study, the Raosoft sample size calculator designed specifically for population surveys was used. Assuming that between 50000 registered HCWs, 40000 of them are actively practicing at the health facilities level, a 95% confidence level was used and an absolute error was estimated to be 5%. All previous information was used to calculate the sample size for this study which yielded the least required sample size of 381 participants. The required sample size was achieved at an early stage before the closure of response acceptance (January 1^st^, 2021).

### Data collection

An online questionnaire using a Google form was emailed to governmental and private hospital directors. Then, designated focal persons working in Lebanese hospitals were contacted via phone call and notified about the survey and its purpose. Upon their agreement to participate, the link of the study was sent through “WhatsApp” to the designated focal person (infection control personnel) who was requested in his turn to disseminate it among other HWCs facilities. This link included a brief introduction to the background, the objective of the survey, and instructions for filling the questionnaire. HCW is defined as “any regulated health professionals and any staff member, or other essential caregivers currently working in a health care organization, including cleaning staff, food services staff, and other administrative staff”. Participants were identified via the infection control personnel at the hospital. All HWCs, working in Lebanese hospitals in different provinces in Lebanon and who agreed to participate in the study, were eligible for participation.

### Ethical considerations

An electronic informed consent was obtained for each participant. They were reassured that their participation is voluntary and that they were free to withdraw at any time. In addition, all information were gathered anonymously and handled confidentially. The questionnaire was collected only in subjects who expressed consent for study participation. As individual participants cannot be identified based on the presented material, this study caused no plausible harm or stigma to participants. The study design respected the participant’s confidentiality and assured adequate protection of study participants, and neither included clinical data about patients nor configured itself as a clinical trial. Hence, this study was exempted from ethical approval by the Ministry of Public Health.

### Statistical analysis

The data were analyzed using the statistical software SPSS (Statistical Package for Social Sciences), version 22.0. Descriptive statistics were reported using frequency with percentages for categorical variables. Bivariate analysis was performed to examine factors associated with the dependent variable (HCW’s willingness to vaccinate) and the demographic variables in addition to the domains of HBM. The relation between nominal variables was tested using the chi-squared test. The variables in bivariate analysis with p-value < 0.2 were entered into multivariable logistic regression. Adjusted odds ratio and their 95% confidence intervals were reported. The final logistic regression model to determine the predictors of willingness to vaccinate was reached after confirming the adequacy of the data using the Hosmer and Lemeshow test. The level of statistical significance was set at a p-value < 0.05.

## Results

### Baseline characteristics of the study participants

A total of 1800 HCWs completed the survey. Table 1 displayed the sociodemographic and clinical characteristics of the study participants. The majority of participants were female 1209 (67.1%) and 904(50.2%) were aged between (30–49) years old. About two-thirds of the HCWs were married 1127(62.6%) and reside in urban areas 1105(61.4%). About half of the participants 880(48.9%) were nurses and 1045(58.1%) were frontline workers Most of the surveyed HCWs had a good health status 1465(81.4%) and were covered by public insurance 1433(79.60%). Only 396(22%) of them suffered from comorbidities and 377(20.9%) of them reported a history of COVID-19 infection. The majority of respondents 1654(91.9%) had a colleague infected by COVID-19.

**Table 1:** Baseline characteristics of the participants (N = 1800).

	n	%
**Gender**		
Male	593	32.9%
Female	1209	67.1%
**Age**		
18–29 years	651	36.2%
30–49 years	904	50.2%
>50 years	245	13.6%
**Marital status**		
Married	1127	62.6%
Unmarried	673	37.4%
**Urbanicity**		
Rural	695	38.6%
Urban	1105	61.4%
**Occupation**		
Physician	382	21.2%
Nurse	880	48.9%
Pharmacist	124	6.9%
Administrative	206	11.4%
Others (Midwife, Lab technician…)	208	11.6%
**Health status**		
Fair and Below	335	18.60%
Good and above	1465	81.40%
**Suffering from comorbidities**		
No	1404	78.0%
Yes	396	22.0%
**Health coverage**		
Public	1433	79.60%
Private	220	12.20%
None	147	8.20%
**Frontline Worker**		
No	755	41.9%
Yes	1045	58.1%
**Previously diagnosed with COVID-19**		
No	1423	79.1%
Yes	377	20.9%
**Family member/friend ever diagnosed with COVID-19**		
No	1087	60.40%
Yes	713	39.60%
**Colleague ever diagnosed with COVID-19**		
No	146	8.10%
Yes	1654	91.90%
**Total**	1800	100%

N: frequency, %: Percentage.

### Past behavior toward vaccination

Only 45.4% of surveyed HCWs have received the influenza vaccine in the past season whereas 67.3% of them declared that they got vaccinated against influenza during the current season. Only 18.9% of them had reported that they refused a type of vaccine other than influenza in the past.

### HCW’s willingness to receive the COVID-19 vaccine

**Fig 1:**
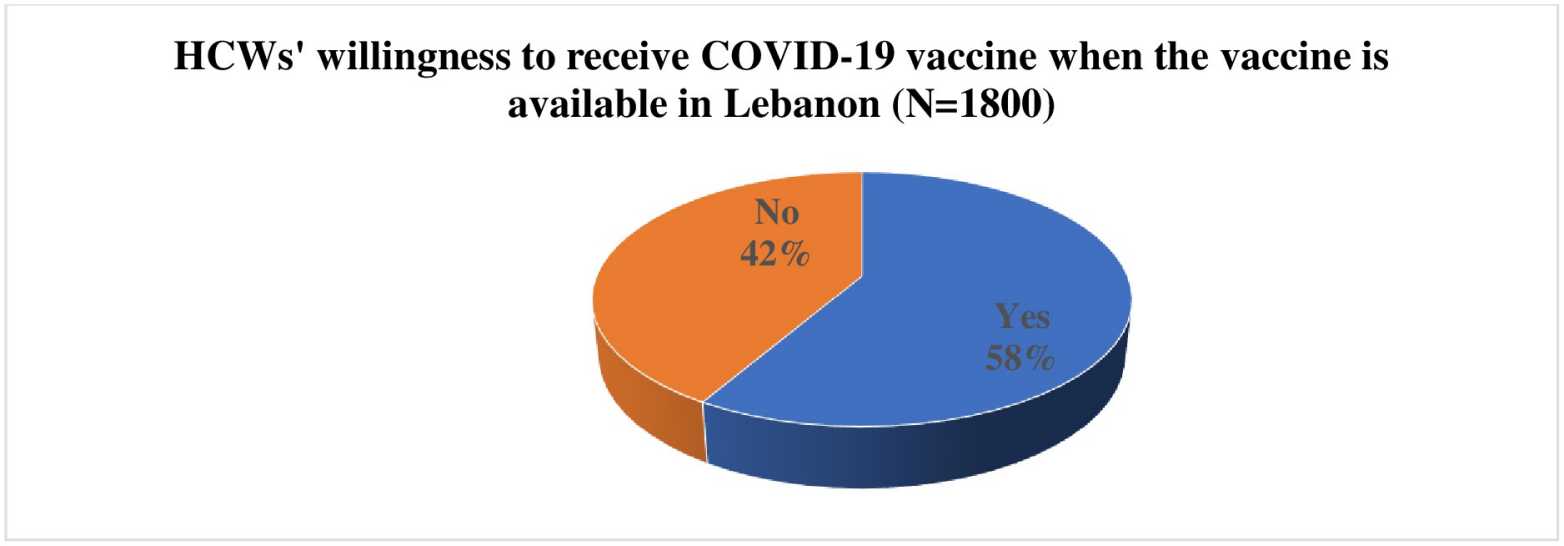
HCW’s willingness to receive the COVID-19 vaccine once available in Lebanon.

Fig 1 showed the willingness of the study participants to take the vaccine. Of the total, 58% of surveyed HCWs expressed their willingness to receive the COVID-19 vaccine once it is available in Lebanon (Fig 1).

### HCWs’ willingness to receive COVID-19 vaccine by province

**Fig 2:**
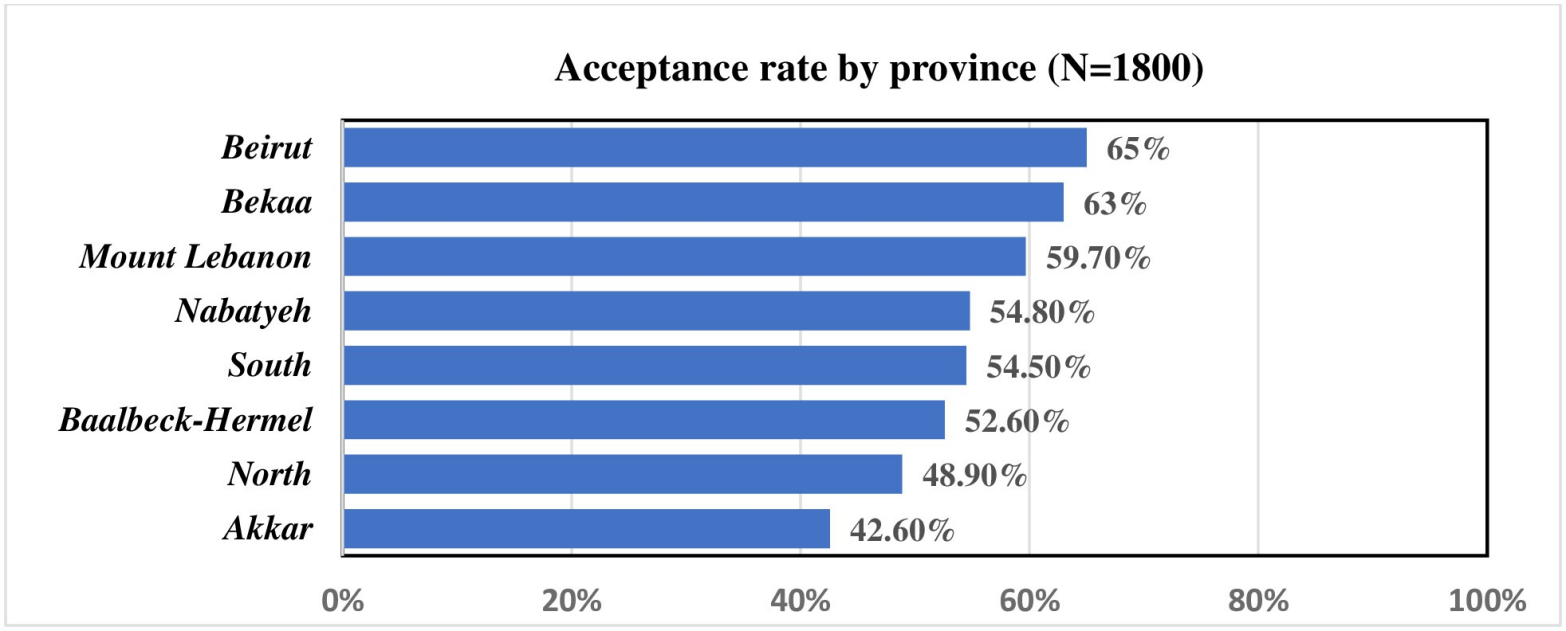
HCWs’ willingness to receive COVID-19 vaccine by province.

Variance in the acceptance rate of COVID-19 vaccine was identified also between geographical areas where Akkar reported the lowest rate (42.6%), compared to Beirut (Capital) that ranked the top (Fig 2).

### Health belief model constructs among HCWs

Table 2 displayed the dimensions of HBM as perceived by HCWs.

**Table 2:** HBM items: Perceived susceptibility to COVID-19, perceived severity and seriousness, perceived benefits, perceived barriers, cues of action, and health motivation.

	Disagree	Neutral	Agree
**Perceived susceptibility to COVID-19**			
I am susceptible to being infected due to my occupational exposure	46(2.6%)	197(10.9%)	1557(86.5%)
There is a great chance to get infected by COVID-19	119(6.6%)	430(23.9%)	1251(69.5%)
Healthy people can get COVID-19	47(2.6%)	104(5.8%)	1649(91.6%)
My health status makes me more susceptible to contract COVID-19	897(49.8%)	526(29.2%)	377(20.9%)
I believe that I can protect myself against COVID-19 better than other people	242(13.4%)	614(34.1%)	944(52.4%)
**Perceived severity and seriousness**			
COVID-19 can make some people very ill and can be fatal	24(1.3%)	140(7.8%)	1636(90.9%)
COVID-19 is more serious than seasonal influenza	81(4.5%)	178(9.9%)	1541(85.6%)
if I get COVID-19, I will be very sick	345(19.2%)	927(51.5%)	528(29.3%)
If I get COVID-19, I might require hospitalization	415(23.1%)	832(46.2%)	553(30.7%)
If I get COVID-19, I might die	520(28.9%)	890(49.4%)	390(21.7%)
**Perceived benefits**			
Vaccination is a good idea because it makes me feel less worried about catching COVID-19	421(23.4%)	464(25.8%)	915(50.8%)
Vaccination decreases my chance of getting COVID-19 or its complications	326(18.1%)	513(28.5%)	961(53.4%)
When I get vaccinated, I protect my patients, family, and friends from infection	330(18.3%)	540(30%)	930(51.7%)
When I get vaccinated, the whole community benefits by preventing the spread of COVID-19	285(15.8%)	536(29.8%)	979(54.4%)
COVID-19 vaccination is an effective way to prevent and control COVID-19	251(13.9%)	541(30.1%)	990(55%)
High vaccination coverage globally is required to stop COVID-19 pandemic	225(12.5%)	571(31.7%)	1004(55.8%)
**Perceived barriers**			
Concerned about the novelty of vaccine (not used before)	151(8.4%)	386(21.4%)	1263(71.1%)
Concerned about the side effects of COVID-19 vaccine	134(7.4%)	330(18.3%)	1336(74.2%)
Concerned about the efficacy of COVID-19 vaccine	193(10.7%)	342(19%)	1265(70.3%)
Concerned about the safety of COVID-19 vaccine	156(8.7%)	371(20.6%)	1273(70.7%)
Concerned about the cost of COVID-19 vaccine (willingness to pay)	429(23.8%)	539(29.9%)	832(46.2%)
Concerned about the accessibility of COVID-19 vaccines	529(29.6%)	563(31.3%)	708(39.3%)
Concerned about the availability of COVID-19 vaccine in limited quantities	239(13.3%)	475(26.4%)	1086(60.3%)
Concerned about the halal nature of the available vaccination	862(47.9%)	546(30.3%)	392(21.8%)
Concerned about the reliability of the manufacturer and the supply source	329(18.3%)	573(31.8%)	898(49.9%)
Concerned about the Lebanese health system and the strategy of the vaccine’s distribution	157(8.7%)	452(25.1%)	1191(66.2%)
Concerned about vaccine mode of administration (needles use…)	709(39.4%)	518(28.8%)	573(31.8%)
Concerned about vaccine frequency (number of doses required….)	510(28.3%)	559(31.1%)	731(40.6%)
Concerned about immunity duration (how much time I will be protected)	156(8.7%)	493(27.4%)	1151(63.9%)
**Cues of action**			
COVID-19 vaccine uptake once reliable information are available	326(18.1%)	317(17.6%)	1157(64.3%)
COVID-19 vaccine uptake if it is recommended by the health facilities	515(28.6%)	445(24.7%)	840(46.7%)
COVID-19 vaccine uptake if it is recommended by the health authorities	549(30.5%)	393(21.8%)	858(47.7%)
COVID-19 vaccine uptake if it is recommended by the media	1324(73.6%)	357(19.8%)	119(6.6%)
COVID-19 vaccine uptake if it is recommended by my work	741(41.2%)	479(26.6%)	580(32.2%)
COVID-19 vaccine uptake if it is taken by many in the public	765(42.5%)	531(29.5%)	504(28%)
**Self-motivation**	**Never**	**Occasionally**	**All the time**
I frequently do things on my own to improve my health	61(3.4%)	388(21.6%)	1351(75.1%)
I have the recommended yearly physical examinations in addition to visits related to illness	86(4.8%)	339(18.8%)	1375(76.4%)

All results are presented in terms of frequency and percentage.

In terms of susceptibility, the majority of participants (86.5%) perceived themselves as susceptible to contracting COVID-19 due to their occupational exposure and 91.6% of them agreed that healthy people can get COVID-19. Only half of them (49.8%) declared that they can protect themselves better than other people. However, 20.9% of respondents reported that their health status makes them more susceptible to getting infected by COVID-19.

In terms of the severity and seriousness of COVID-19 infection, the bulk of surveyed HCWs perceived the severity of COVID-19 infection and agreed that it could be fatal for some people. Furthermore, the majority (85.6%) considered that COVID-19 is more serious than influenza. However, only 29.3% of participants thought that they will be very ill if they contracted COVID-19, 30.7% stated that they may need hospitalization and 21.7% pondered that they might die in case they contracted COVID-19.

Regarding the perception of benefits, more than 50% of surveyed HCWs perceived the benefits of the COVID-19 vaccine since it makes them less worried about COVID-19 and decreases the likelihood of developing complications from COVID-19. In addition, they reported that if they get the vaccine, they could protect their close environment (patients, family, and friends) from the infection and the whole community in a wider range. Lastly, they endorsed the ability of vaccination to control effectively the pandemic and agreed on the need for high vaccination coverage to stop the pandemic.

Concerning perceived barriers of COVID-19 vaccination, the major barriers perceived by HCWs were the side effects of the COVID-19 vaccines (74.2%), the novelty of vaccines (71.1%), the safety of the vaccine (70.7%), the efficacy of the vaccine (70.3%), the limited availability of vaccines (60.3%), and the strategy adopted by the health system for the distribution of vaccines (66.2%), the duration of the acquired immunity (63.9%). Similarly, HCWs perceived vaccine efficacy (85.3%), vaccine safety (72.6%), and side effects of vaccines as main barriers.

In terms of cues to action, receiving adequate and reliable information about vaccines (64.3%) and recommendation of the vaccine from health facilities (46.7%) and health authorities (47.7%), and were found the main cues that promote willingness to get COVID-19 vaccine among Lebanese HCWs. Only 6.6% of the surveyed HCWs could receive the COVID-19 vaccine if this was recommended by the media.

More than a third-quarter of surveyed HCWs did things to improve their health (sport, diet…) and complied with the recommended yearly physical examination and follow-up related to their health status.

### Knowledge about the COVID-19 vaccine

Table 3 displayed the HCWs responses to the knowledge items related to the vaccine. Approximately two-quarters of them (76.8%) were found to be knowledgeable about the basic information related to COVID-19 vaccines. However, only half of the participants were knowledgeable about the mode of action of RNA and DNA vaccines that give our bodies the genetic code it needs to allow our immune system to produce the antigen on its own. However, 80.4% of respondents emphasized the importance of compliance with recommended preventive measures as well COVID-19 vaccination for young people.

**Table 3:** Knowledge items related to the COVID-19 vaccine.

	Correct	Incorrect	I Don’t Know
	n (%)	n (%)	n (%)
Vaccines are effective in combating highly contagious diseases ^c^	1307(72.6%)	97(5.4%)	396(22%)
Traditionally, vaccines create immunity by introducing a weak form of an infectious agent that allows the immune system to build a memory against this agent ^c^	1358(75.4%)	44(2.4%)	398(22.1%)
The RNA and DNA vaccines give our bodies the genetic code it needs to allow our immune system to produce the antigen on its ^c^	972(54%)	83(4.6%)	745(41.4%)
Covid-19 vaccines are being developed as quickly as possible, but they were required to receive the necessary regulatory licenses ^c^	740(41.1%)	157(8.7%)	903(50.2%)
The flu vaccine protects against covid-19^F^	1283(71.3%)	78(4.3%)	439(24.4%)
People with chronic diseases and the elderly are more likely to have the disease and its complications, so they should get the vaccine ^c^	1334(74.1%)	135(7.5%)	331(18.4%)
Young people are healthy and therefore do not need to follow preventive measures and get the vaccine to protect themselves against Covid-19^F^	1447(80.4%)	138(7.7%)	215(11.9%)
Until the readiness and the availability of the COVID-19 vaccine, we cannot do anything to tackle the disease ^F^	1185(65.8%)	331(18.4%)	270(15%)

^c^ correct statement,

^F^ false statement, n frequency, % percentage.

### Reliability of sources of information as perceived by HCWs

**Fig 3:**
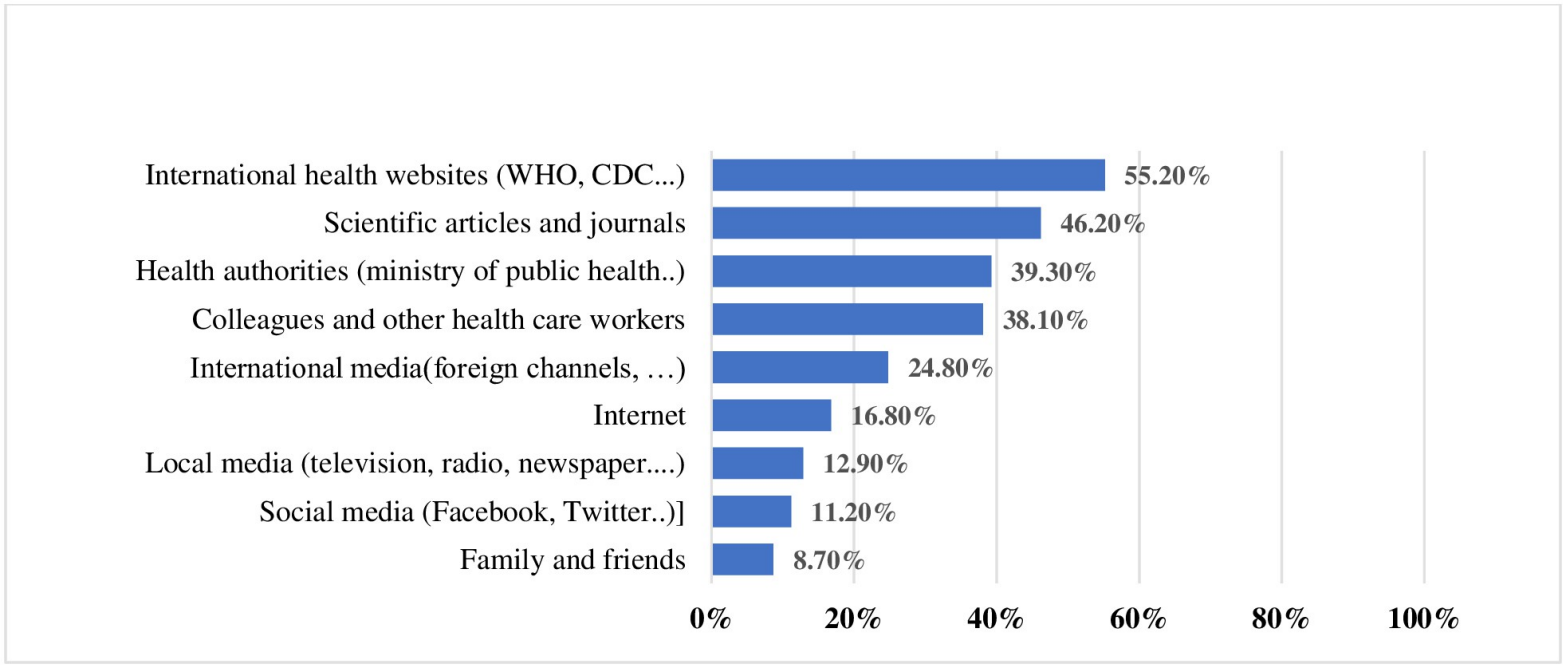
Reliable sources of information about COVID-19 vaccines as perceived by HCWs.

As for reliable sources of information on vaccines, the most reliable sources ranked by HCWs were the international health websites such as WHO and CDC followed by scientific articles (46.2%), and health authorities (39.3%) while family and friends were listed the least trusted information sources (Fig 3).

### Factors associated with COVID-19 vaccine acceptance among HCWs

Table 4 summarized the multivariable logistic regression of the factors associated with the willingness to receive the COVID-19 vaccine. HCWs who were male (aOR = 1.99, 95% CI (1.41–2.80)), working in the frontlines (aOR = 1.61, 95% CI (1.17–2.21), and those who have received influenza vaccination for the current year (aOR = 1.38, 95% CI(0.99–1.92)) were more willing to get the COVID-19 vaccine. However, factors such as living in rural areas (aOR = 0.61, 95% CI (0.44–0.84)), and being previously diagnosed with COVID-19 (aOR = 0.66, 95%CI (0.45–0.96) were found negatively associated with vaccine acceptance. In terms of health beliefs items, concerns related to the novelty of vaccine (aOR = 0.42, 95% CI (0.25–0.71)), side effects/vaccine safety (aOR = 0.41, 95% CI (0.23–0.73), reliability of manufacturer (aOR = 0.43, 95% CI (0.30–0.63) and), and the number of required doses (aOR = 0.58, 95% CI (0.40–0.84) were also negatively associated with the willingness to get vaccinated against COVID-19. Remarkably, concerns such as the limited accessibility (aOR = 1.68, 95% CI (1.14–2.47), and availability of vaccines (aOR = 2.16, 95% CI (1.46–3.20)were associated with an increased likelihood of willingness to receive the COVID-19 vaccine. With regards to cues of action, receiving reliable and adequate information about the vaccine (aOR = 1.98, 95% CI (1.36–2.88)), recommendation by health authorities (aOR = 1.93, 95% CI(1.33–2.81)), and recommendations from health facilities (aOR = 2.68, 95% CI(1.80–3.99)) were also positively associated with vaccine acceptance, whilst recommendation by a family member (aOR = 0.47, 95% CI(0.28–0.81)) was negatively associated with the willingness to get vaccinated. Lastly, perception of COVID-19 vaccine benefits by HCWs in terms of protecting them and their close contacts (patients, family members, and friends) from COVID-19 infection (aOR = 4.21, 95% CI (2.78–7.11) was associated with an increased likelihood of vaccine uptake.

**Table 4:** Factors associated with the willingness to take COVID-19 vaccines among HCWs in Lebanon (N = 1800).

	aOR	95% CI		P-value
		Lower	Upper	
Gender (Male vs female)	1.99	1.41	2.80	<0.001
Urbanicity (rural vs urban)	0.61	0.44	0.84	0.002
Frontline workers (yes vs No)	1.61	1.17	2.21	0.003
Previously diagnosed with COVID-19 (yes vs No)	0.66	0.45	0.96	0.032
Received flu vaccination this year (yes vs No)	1.38	0.99	1.92	0.047
**Perceived barriers**				
Concerned about the novelty of the vaccine (agree vs disagree/neutral)	0.42	0.25	0.71	0.001
Concerned about the safety/side effects of the vaccine (agree vs disagree/neutral)	0.41	0.23	0.73	0.002
concerned about the accessibility of COVID-19 vaccines (agree vs disagree/neutral)	1.68	1.14	2.47	0.009
Concerned about the availability of COVID-19 vaccine in limited quantities (agree vs disagree/neutral)	2.16	1.46	3.20	<0.001
Concerned about the reliability of the manufacturer and the supply source (agree vs disagree/neutral)	0.43	0.30	0.63	<0.001
Concerned about the number of required doses (agree vs disagree/neutral)	0.58	0.40	0.84	0.003
**Benefits of vaccine** (protecting patients, family, and friends from infection once vaccinated)	4.21	2.78	7.11	<0.001
**Cues of action**				
COVID-19 vaccine uptake once reliable information are available (agree vs disagree/neutral)	1.98	1.36	2.88	<0.001
COVID-19 vaccine uptake if it is recommended by the health authorities (agree vs disagree/neutral)	1.93	1.33	2.81	<0.001
COVID-19 vaccine uptake if it is recommended by the health facilities (agree vs disagree/neutral)	2.68	1.80	3.99	<0.001

aOR: adjusted OR, 95% CI 95% confidence interval, P-value <0.05 is considered significant.

## Discussion

To mitigate the increasing threats and challenges presented by the COVID-19 pandemic, Lebanon plans to begin the first round of immunization against COVID-19 in the first quarter of this year. This plan slated HCWs and the elderly to be the first groups to receive the COVID-19 vaccine. Since HCWs are at high occupational risk for COVID-19 infection and will be lately responsible for administering and recommending vaccines to their patients and the public, it’s critical to assess their willingness to get vaccinated once the COVID-19 vaccine is available in Lebanon. To the best of our knowledge, this is the first large national study to assess the COVID-19 vaccine uptake among Lebanese HCWs and to explore the factors that influence HCWs’ willingness to get the COVID-19 vaccine.

The study’s main findings were that roughly 58% of surveyed participants were willing to receive the COVID-19 vaccine once it is available. Our results showed that male HCWs, frontline workers, and those who have received influenza vaccination were more likely to receive the vaccine. However, HCWs living in rural areas, and being previously diagnosed with COVID-19 were less likely to get the COVID-19 vaccine. The majority of the HBM domains were considerably associated either positively or negatively with vaccine uptake. Respondents who perceived the vaccine as conferring benefits, and received cues to action were significantly more likely to accept the vaccine. However, respondents who expressed concerns about the novelty, side effects, safety, reliability of the manufacturer, and the required number of doses to be immune were less likely to get the COVID-19 vaccine. Remarkably, limited accessibility and availability were positively associated with higher intent to vaccinate.

The current study’s findings concerning vaccine acceptance are in line with those of prior studies conducted in other parts of the world. A cross-sectional conducted in the Kingdom of Saudi Arabia (KSA) prior to the launch of the vaccine campaign revealed that 52.6% of HCWs indicated a willingness to receive a vaccine as soon as possible, while 35.6% reported preferring to wait for a few months before receiving one, and 11.8% indicated that they would never agree to receive any potential vaccine [23]. A systematic review regarding the acceptability of the COVID-19 vaccine found that the proportion of HCWs that intent to accept the COVID-19 vaccination was 55.9% [24]. The moderate rate of COVID-19 vaccine acceptance reported in our study should trigger public health officials to target these groups with campaigns to enhance their vaccine confidence and acceptability.

In terms of socio-demographic characteristics, a gender difference was revealed in the willingness rate to receive the COVID-19 vaccine with males HCWs being significantly more likely to receive the vaccine than females. A study conducted in New Zealand showed that two-thirds of women were willing to be vaccinated compared to three-quarters of men [2]. As women are anticipated to make up 70% of the global COVID-19 healthcare workforce, it is crucial to address this disparity to optimize vaccination for both genders. Furthermore, our results showed geographical variation in HCWs vaccine acceptance between rural and urban areas. Such a pattern was expected since rural areas have usually high vaccine hesitancy compared with urban ones.

As expected, frontline workers were more likely to receive the vaccine compared to their counterparts who were not. This could be explained by the fact that frontline HCWs perceived themselves as more susceptible to getting COVID-19 due to their occupational exposure. Our results were in line with a study conducted in the USA that revealed a high vaccine uptake among HCWs involved in direct patient care [14].

Having a personal history of COVID-19 infection was negatively associated with the willingness to vaccinate. This could be explained by the fact that most of the previously infected people consider themselves naturally immune, hence they refuse to get vaccinated. It is worth noting that a high proportion of survey HCWs reported previous SARS-COV-2 infection compared to other countries. For example, a study conducted among 16,912 HCWs in Qatar revealed that 10.6% of HCWs were positive for COVID-19 [25]. Another study conducted in India showed a prevalence of COVID-19 infection of 11% among HCWs [26]. Since infection of HCWs was linked to lack of sufficient personal protective equipment (PPEs) at facilities, and poor compliance to infection control measures, hence looking in-depth into the factors associated with COVID-19 among HCWs is recommended for ensuring their safety. Our results also revealed that HCWs with a history of prior influenza vaccination were more likely to get vaccinated against COVID-19. This could be explained that people who have had influenza vaccination pay greater attention to respiratory disease prevention and have a better understanding of vaccines. In comparison to the previous season, when less than half of the surveyed HCWs received their influenza vaccine, the current season’s influenza vaccination coverage has increased by more than 20 percent. This increase could be due to the special attention accorded to the influenza vaccination during this season where the SARS-Cov2 and the influenza viruses co-circulate. In such a context, public health experts have stressed the importance of getting vaccinated against influenza to avoid possible dual infection. Such findings also revealed a positive attitude toward vaccination.

Despite some key domains of HBM such as perceived severity and perceived susceptibility were not found with COVID-19 vaccine acceptance, the majority of our participants considered themselves susceptible to getting COVID-19 due to their occupational exposure. Our results were consistent with a risk perception assessment of COVID-19 conducted among Portuguese HCWs which found that 54.9% perceived themselves at a high likelihood of becoming infected [27]. Another study conducted in the USA showed that HCWs, particularly nurses, have a higher prevalence of SARS-CoV-2 infection than non-healthcare workers, according to researchers at Rutgers [28]. The absence of association between perceived severity and vaccine uptake could be explained by the low perception of severity among HCWs in terms of complications, hospitalization, and fatality. This stressed the importance of unveiling the potential complications of COVID-19 recorded among COVID-19 cases.

As for barriers, the main concerns expressed by HCW’s that could hamper their willingness to vaccinate against COVID-19 were their concerns about novelty, side effects, efficacy, and vaccine safety. There are vigorous testing trials in place to warrant that approved COVID-19 vaccines are both effective and safe [29]. However, vaccination, like all kinds of therapies could have adverse events. Addressing the extent of those risks presents the biggest challenge in the development of the COVID-19 vaccine. Among the cited barriers, the novelty of the vaccine, possible side effects induced by the vaccine, the reliability of the manufacturer, and the trust in the vaccine’s source in addition to the number of doses required to get immune as well, were found negatively associated with the vaccine uptake in the current study. Our findings supported the assumption that trust in vaccine manufacturers and sources played an important role in explaining the pattern of vaccine uptake [30]. Because of the huge demand for the COVID 19 vaccines, several new companies that were not well recognized before entered the market [31]. However, the limited evidence regarding vaccine safety and effectiveness combined with a large number of new vaccines manufacturers and novel technologies all at once would increase vaccines recipients’ skepticism. This will, in turn, potentially limit their vaccine uptake. To overcome such obstacles, governments should act proactively, and incorporate information on their chosen vaccine producer in advance (s).

A peculiar finding in our study was that HCWs who conceived limited availability and accessibility of COVID-19 vaccine as barriers were more likely to get vaccinated. This could be explained by a learning pattern of human nature that usually desires unreachable things.

In terms of benefits, our respondents mentioned that the main benefits of getting vaccinated against COVID-19 include decreasing their fears about catching COVID-19, decreasing their chance of getting infected or presenting complications, protecting their patients and family members, and preventing the spread of COVID-19 at the community level. This was consistent with the findings of a study conducted among the Chinese population who perceived the same benefits [32]. In terms of benefits, surveyed HCWs who perceived the vaccine benefits in protecting their close environment from COVID-19. Our results were concordant with a study conducted in France that stresses the importance of communicating the benefits of vaccines in reducing COVID-19 vaccine hesitancy [29].

With regards to cues of action, the recommendation of the COVID-19 vaccine from the health authorities and the health facilities stood out as the most important cues affecting positively the intent to vaccinate. As for these recommendations, a substantial discrepancy was recorded between countries, with China again having the highest proportion of positive responses (83.7%) compared to Russia that had the highest proportion of negative responses in terms of acceptance of their employer’s recommendation [33].

Furthermore, having reliable, sufficient, and adequate information regarding the vaccine also turn out to be an important driver of vaccine acceptance. These observations highlight the evidence-based design of vaccine promotion campaigns tailored for the context of the HCWs concern.

Consistently to other studies that demonstrated the capability of the HBM constructs in predicting behaviors related to influenza vaccination [34]. Our findings suggested that the domains of HBM could be used to elucidate vaccine uptake behavior.

### Limitations of the study

Several limitations of this study should be acknowledged. Firstly, this is a cross-sectional survey and we could not establish a cause-and-effect relationship between the independent factors and the outcome. They could however be used in the prediction of COVID-19 vaccine acceptance. Also, this survey examined the HBM constructs among Lebanese HCWs, and the generalizability of its findings to other settings should be cautious. Selection bias is possible due to the snowball technique that was used to collect data. Secondly, our study relies on HCWs’ self-reported information, which may be a threat to information bias. In addition, self-reported information could be associated with recall bias about past behaviors. On other hand, participants’ responses may be influenced by social desirability and then intentionally modified to meet the norms. In addition, a selection bias is possible since the survey was online and relies on the availability and accessibility to the internet. The snowball sampling technique used for data collection may also lead to selection bias. Finally, the study was conducted before the vaccine was available and information on vaccine efficiency and safety at that point were not accurate. Thus, the actual vaccine uptake against COVID-19 could change once the vaccine becomes available.

## Conclusion

The moderate acceptance rate of the COVID-19 vaccine among HCWs could have broader extents such as triggering a ripple effect in the general public. Our findings also highlighted the significance of the recommendation of vaccines by the government and the health facilities on improving vaccine uptake among HCWs. However, vaccine acceptance was found impaired by concerns on novel vaccination approaches and manufacturers’ reliability. Understanding and pointing out these factors that drive moderate vaccine uptake via appropriate interventions are required. This encompassed addressing key concerns and increasing awareness about the COVID-19 vaccine through targeting messages to reach a higher vaccination rate. Otherwise, there is an additional chance of mass hesitancy among the general population when the COVID-19 vaccine becomes available.

## Supporting information

S1 File (DOCX)
