# Supplementary material for: Determinants of acceptance of Coronavirus disease-2019 (COVID-19) vaccine among Lebanese health care workers using health belief model
Source: PLoS One. 2022 Feb 22;17(2):e0264128. doi: 10.1371/journal.pone.0264128 (PMC8863223; doi:10.1371/journal.pone.0264128)
Supplement: S1 File — (DOCX) [file pone.0264128.s001.docx]

**Determinants of acceptance of COVID-19 vaccine among Lebanese Health Care Workers using Health Belief Model**

Immunization programs are only successful when there are high rates of acceptance and coverage. To accomplish this, it is critical to understand health care workers’ risk perceptions about COVID-19, acceptance of a COVID-19 vaccine, and conﬁdence in media sources, speciﬁcally those used to obtain information about the COVID-19 pandemic. You are cordially invited to fill this survey which will take less than 10 minutes to complete. Participation in this survey is completely voluntary and all of your responses are anonymous. This survey aims to understand the determinants of acceptance of the COVID-19 vaccine among healthcare workers in Lebanon. If you have any questions related to this survey, or difficulty in accessing the site, please contact: Dalal Youssef (dalalyoussef.esu@gmail.com or 01/830300 extension 437)

Your participation is highly appreciated!

**Section 1: Baseline characteristics of the study participants**

| **Gender** |
| --- |
| Male |
| Female |
| **Age** |
| 18-29 years |
| 30-49 years |
| >50 years |
| **Marital status** |
| Single |
| Married/Engaged |
| Other (Divorced or Widowed) |
| **Urbanicity** |
| Rural |
| Urban |
| **Occupation** |
| Physician |
| Nurse |
| Pharmacist |
| Administrative |
| Others (Midwife, Lab technician…) |
| **Health status** |
| Fair and Below |
| Good and above |
| **Presence of underlying health condition** |
| Yes |
| No |
| **Presence of child at home** |
| Yes |
| No |
| **Presence of elderly people at home** |
| Yes |
| No |
| **Health insurance** |
| Public |
| Private |
| None |
| **Working in frontline in the response to COVID-19** |
| Yes |
| No |
| **Personal history of COVID-19 diagnosis** |
| Yes |
| No |
| **Family member/friend ever diagnosed with COVID-19** |
| Yes |
| No |
| **Colleague ever diagnosed with COVID-19** |
| Yes |
| No |

**Section 2: HBM**

| 2.1 | **Perceived susceptibility to COVID-19** | | | |
| --- | --- | --- | --- | --- |
| S1 | I am susceptible of getting infected due to my occupational exposure | **Disagree** | **Neutral** | **Agree** |
| S2 | There is a great chance to get infected by COVID-19 in the next coming months especially during winter | **Disagree** | **Neutral** | **Agree** |
| S3 | Healthy people can get COVID-19 | **Disagree** | **Neutral** | **Agree** |
| S4 | My health status makes me more susceptible to contract COVID-19 | **Disagree** | **Neutral** | **Agree** |
| S4 | I believe that I can protect myself against COVID-19 better than other people | **Disagree** | **Neutral** | **Agree** |
| 2.2 | **Perceived severity and seriousness** | | | |
| Sev1 | Although for most people, COVID-19 causes mild illness, it makes some people very ill and can be fatal | **Disagree** | **Neutral** | **Agree** |
| Sev2 | I think COVID-19 is more serious than seasonal influenza | **Disagree** | **Neutral** | **Agree** |
| Sev3 | I will be very sick if I get COVID-19 | **Disagree** | **Neutral** | **Agree** |
| Sev4 | If I get COVID-19, I might require hospitalization | **Disagree** | **Neutral** | **Agree** |
| Sev5 | If I get COVID-19, I might die | **Disagree** | **Neutral** | **Agree** |

| **2.3** | **Perceived benefits** | | | |
| --- | --- | --- | --- | --- |
| I1 | Vaccination is a good idea because it makes me feel less worried about catching COVID-19 | Disagree | Neutral | Agree |
| I2 | Vaccination decreases my chance of getting COVID-19 or its complications | Disagree | Neutral | Agree |
| I3 | Vaccines are considered between the most tested and safe medical products | Disagree | Neutral | Agree |
| I4 | When I get vaccinated, I protect my patients, family and friends from infection | Disagree | Neutral | Agree |
| I5 | When I get vaccinated, the whole community benefits by preventing the spread of COVID-19 | Disagree | Neutral | Agree |
| I6 | COVID-19 vaccination is an effective way to prevent and control COVID-19 | Disagree | Neutral | Agree |
| I7 | High vaccination coverage globally is required to stop COVID-19 pandemic | Disagree | Neutral | Agree |
| **2.4** | **Perceived barriers** | | | |
| B1 | I am concerned about the novelty of vaccine (not used before) | Disagree | Neutral | Agree |
| B2 | I am concerned about the side effects of COVID-19 vaccine | Disagree | Neutral | Agree |
| B3 | I am concerned about the efficacy of COVID-19 vaccine | Disagree | Neutral | Agree |
| B4 | I am concerned about the safety of COVID-19 vaccine | Disagree | Neutral | Agree |
| B5 | I am concerned about the cost of COVID-19 vaccine (willingness to pay) | Disagree | Neutral | Agree |
| B6 | I am concerned about the accessibility of COVID-19 vaccines (geographical distribution of sites) | Disagree | Neutral | Agree |
| B7 | I am concerned about the availability of COVID-19 vaccine in limited quantities for limited categories of the population | Disagree | Neutral | Agree |
| B8 | I am concerned whether if the COVID-19 vaccine is halal | Disagree | Neutral | Agree |
| B9 | I am concerned about the reliability of the manufacturer and the source of supply | Disagree | Neutral | Agree |
| B10 | I am concerned about Lebanese health system, and the strategy of distribution of the vaccines | Disagree | Neutral | Agree |
| B11 | I am concerned about vaccine mode of administration (needles use...) | Disagree | Neutral | Agree |
| B12 | I am concerned about vaccine frequency (number of doses required....) | Disagree | Neutral | Agree |
| B13 | I am concerned about immunity duration (how much time I will be protected) | Disagree | Neutral | Agree |

| **2.5** | **Cues of action** |  |  |  |
| --- | --- | --- | --- | --- |
| C1 | I will only take the COVID-19 vaccine if I was given adequate and reliable information about it | Disagree | Neutral | Agree |
| C2 | I will only take the COVID-19 vaccine if the vaccine is recommended by the health facilities | Disagree | Neutral | Agree |
| C3 | I will only take the COVID-19 vaccine if the vaccine was recommended by a family member (relatives...) | Disagree | Neutral | Agree |
| C4 | I will only take the COVID-19 vaccine if the vaccine is recommended by the health authorities | Disagree | Neutral | Agree |
| C5 | I will take COVID-19 vaccine if the vaccine is recommended by the media | Disagree | Neutral | Agree |
| C6 | I will take the COVID-19 vaccine if the vaccine is recommended by my work | Disagree | Neutral | Agree |
| C7 | I will take the COVID-19 vaccine if the vaccine is taken by many in the public | Disagree | Neutral | Agree |
| 2.6 | **Health motivation** |  |  |  |
| H1 | I frequently do things on my own to improve my health | Never | Occasionally | All the time |
| H2 | I have the recommended yearly physical examinations in addition to visits related to illness | Never | Occasionally | All the time |

|  | | | | |
| --- | --- | --- | --- | --- |
| **Section 3: Knowledge about vaccine** | | | | |
| K1 | Vaccines are effective in combating highly contagious diseases ^c^ | True | False | I Don’t Know |
| K2 | Traditionally, vaccines create immunity by introducing a weak form of an infectious agent that allows the immune system to build a memory against this agent ^c^ | True | False | I Don’t Know |
| K3 | The RNA and DNA vaccines give our bodies the genetic code it needs to allow our immune system to produce the antigen on its own ^c^ | True | False | I Don’t Know |
| K4 | Covid-19 vaccines are being developed as quickly as possible, but they were required receive the necessary regulatory licenses ^c^ | True | False | I Don’t Know |
| K5 | The flu vaccine protects against covid-19^F^ | True | False | I Don’t Know |
| K6 | People with chronic diseases and elderly are more likely to have the disease and its complications, so they should get the vaccine ^c^ | True | False | I Don’t Know |
| K7 | Young people are healthy and therefore do not need to follow preventive measures and to get the vaccine in order to protect themselves against Covid-19^F^ | True | False | I Don’t Know |
| K8 | Until the readiness and the availability of COVID-19 vaccine, we cannot do anything to tackle the disease ^F^ | True | False | I Don’t Know |

**Section 4: Reliability of information**

|  |  |  |  |
| --- | --- | --- | --- |
|  | **Little** | **Some** | **Much** |
| Local media (television, radio, newspaper....) |  |  |  |
| International media(foreign channels, …) |  |  |  |
| Health authorities (ministry of public health..) |  |  |  |
| International health websites (WHO, CDC...) |  |  |  |
| Scientific articles and journals |  |  |  |
| Social media (Facebook, Twitter..)] |  |  |  |
| Internet |  |  |  |
| Family and friends |  |  |  |
| Colleagues and other health care workers |  |  |  |
